# Supplementary material for: The causal relationship between the human gut microbiota and pyogenic arthritis: a Mendelian randomization study
Source: Front Cell Infect Microbiol. 2024 Nov 26;14:1452480. doi: 10.3389/fcimb.2024.1452480 (PMC11629706; doi:10.3389/fcimb.2024.1452480)
Supplement: Supplementary file 1 [file DataSheet1.zip › mendelian test/ebi-a-GCST90027699.csv_leave_one_out.pdf]

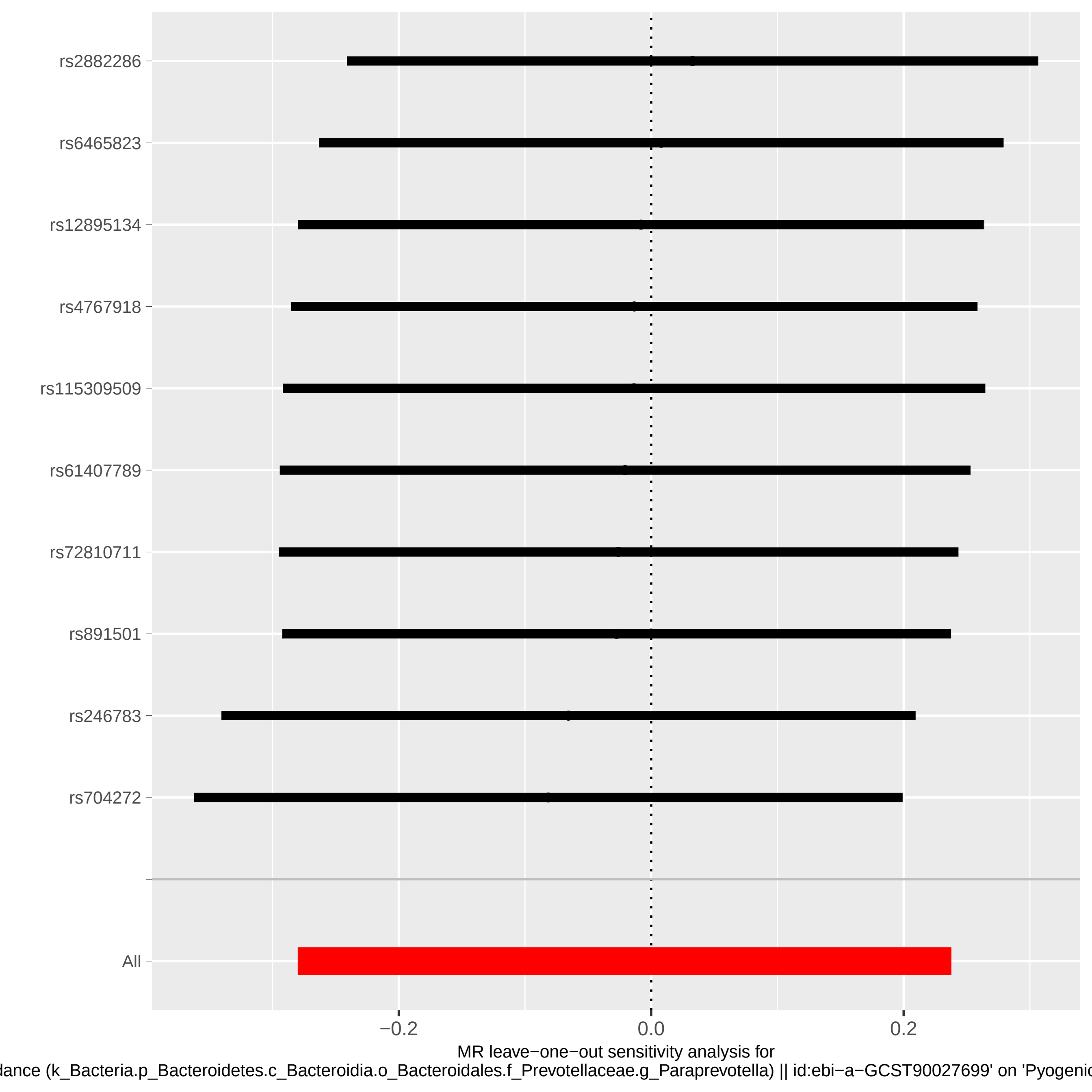
rs2882286
rs6465823
rs12895134
rs4767918
rs115309509
rs61407789
rs72810711
rs891501
rs246783
rs704272
All
−0.2
0.0
0.2
MR leave−one−out sensitivity analysis for
dance (k_Bacteria.p_Bacteroidetes.c_Bacteroidia.o_Bacteroidales.f_Prevotellaceae.g_Paraprevotella) || id:ebi−a−GCST90027699' on 'Pyogenic
